# Discovery That the Veins of the Bat’s Wing (Which Are Furnished with Valves) Are Endowed with Rythmical Contractibility, and That the Onward Flow of Blood Is Accelerated by Such Contraction

**Published:** 1853-07

**Authors:** T. Wharton Jones


*Discovery that the Veins of the Bat's Wing (which are furnished with valves) are endowed with Rythmical Contractibility, and that the onward flow of blood is accelerated by such contraction.* By T. Wharton Jones.—In entering on the investigation of the state of the blood and the blood-vessels in inflammation excited in the web of the bat's wing, I applied myself, in the first place, to the study of the distribution, structure, and endowments of the arteries, capillaries and veins of the part, and of the phenomena of the circulation in them.

I had not observed the circulation under the microscope long, before I was struck with something peculiar in the flow of blood in the veins; I therefore directed my attention to them, and discovered that they contracted and dilated rythmically. Following the veins for some extent in their course, I further discovered them to be provided with valves, some of which completely opposed regurgitation of blood, others only partially.

The cause of the peculiarity in the flow of blood in the veins was thus no longer doubtful; but some continued observation was required before I was able to make out exactly its mode of operation.

The act of contraction of the vein is manifested by progressive constriction of its caliber and increasing thickness of its wall; the relaxation of the vessel, by a return to the former width of caliber and thickness of wall.

The rythmical contractions and dilatations of the veins are, in the natural state, continually going on; but sometimes with greater, sometimes with less rapidity, and sometimes to a greater, sometimes to a less extent. The average number of contractions in a minute, I have found to be ten. I have on some occasions counted only seven or eight, and on other occasions as many as twelve or thirteen. Most usually, the numbers were nine and eleven. The supervening dilatations take place rather more quickly than the contraction. The amount of constriction of one of the larger veins,—one about 1-300th or 1-400th of an inch in width when dilated,—at each contraction of its walls, may be put down at a fourth or fifth of its whole width when in a state of dilatation; I have sometimes estimated it at nearly a third, sometimes at not more than a sixth.

The contractions *centrad* and *distad* of a valve appeared to be simultaneous, as did also the dilatations.

The smaller veins, those of the first and second order, proceeding from the radicals, contract, but not in a very marked manner, and are destitute of valves.

During contraction, the flow of blood in the vein is accelerated. On the cessation of the contraction, the flow is checked, and a tendency to regurgitation of the blood takes place, which brings the valves into play. Where the valves are perfect, the backward movement of the blood is at once stopped by their closure; but where the valves are not complete, the blood regurgitates more or less freely.* But this check to the onward flow of the blood is usually only for a moment or two. Already, even while the vein is in the act of again becoming dilated, the onward flow of blood recommences and goes on, though comparatively slowly, until dilatation is completed and contraction supervenes; whereupon acceleration of the flow takes place as before.

It is to be observed, that in determining the flow of blood in the veins (the phenomena of which I have now described), the action of the heart is concerned as well as the contractions of the veins themselves. It appears to be the heart's action which maintains the onward flow of blood during the dilatation of the vein, whilst it is the contraction of the vein, coming in aid of the heart's action, which causes the acceleration. Sometimes the *vis a tergo* is sufficient to keep up a pretty steady flow in the veins, this being only accelerated at each contraction of these vessels.

The check to the flow of blood in the veins takes place at the completion of the contraction or commencement of the dilatation. The number of checks observable in a minute, therefore, corresponds with the number of contractions. In one case, while an assistant marked the time by a seconds' watch, I observed that a complete valve checked the tendency to regurgitation nine times in a minute; and on counting the number of contractions of the same vessel, I found them also nine in a minute. In another case, eleven checks and eleven contractions were counted; and so on repeatedly. Though I quote these little experiments, I would remark that, after some practice in the observation, the eye is quite able to take in at one glance the succession and relations of the two phenomena.

The valves of the veins are composed sometimes of but a single flap, sometimes of two. In the situation of a valve, and *centrad* of the insertion of its flaps, the veins present the usual dilatations or sinuses corresponding to the sinuses of Valsalva at the origin of the pulmonary

* Sometimes, as for example, into a venous branch with an incomplete valve, a retrograde flow of blood takes place from a large vein, at the moment this latter is contracting and propelling its blood onwards.—May 7, 1852.

artery and aorta. These sinuses are best seen when the valve happens to present its flaps edgeways to the observer.

Valves are found close to the entrance of a large branch, but distad of it. They are also found at intermediate parts of the veins. Tracing the veins from radicles to trunks, the first valves I have noticed were at the junction of the second order of veins to form the third.

In watching the circulation, it is interesting to observe the backward eddy of blood-corpuscles into the sinuses of the valves, when the blood issues from the narrow valvular opening into the wide part of the vein beyond.

In structure, the valves are seen to be a reduplication of the clear innermost coat of the vein, with sometimes a pretty evident layer of fibrous tissue intervening.

Each vein is closely accompanied by an artery, a nerve only intervening. The average diameter of a vein is to that of its accompanying artery as about 3 to 2.

The contractility of the arteries is altogether different in its nature from that of the veins. It is *conic contractility, not rythmical.* On the application of pressure over an artery, this vessel may be seen to become constricted, sometimes even to temporary obliteration of its caliber, and that uniformly throughout some extent of its course, both above and below the point where the pressure was applied; or, the constriction is greater or less at intervals, so that the vessel presents a varicose appearance. This tonic contraction of the arteries of the bat's wing does not take place quite so quickly as the same phenomenon in the frog's web, and, ordinarily, continues a longer time.

The pulsation of a vein so affects its accompanying artery as to push the latter, as a whole, to and fro. That the movement of the artery referred to is really owing to this cause, and not to any pulsation or rythmical contraction and dilatation of its own walls, is evident from this, that the movements are synchronous with the contractions and dilatations of the vein, and that *both sides of the artery move in the same direction*, not approximating and receding from each other, so as to constrict or dilate the caliber, as in the case of the vein.

I have not been able to observe unequivocal evidences of tonic contractility of veins in addition to their rythmical contractility. When pressure is, at the same time, applied over the vein as well as the artery, the vein is not found to become tonically constricted in the same manner as the artery, upward and downward. At the place where the vein was pressed on, a mechanical indentation of its wall may perhaps be seen. And, in addition to this, there may often be observed an appearance of great and abrupt constriction. This appearance, however, is not owing to contraction of the walls of the vein, but to a deposit of a viscid-looking grayish granular lymph within the vessel at the place, obstructing its channel and narrowing the stream of blood. On watching, I have seen portions of this deposit detached and carried away by the stream of blood, with corresponding enlargement of the channel, and again an additional deposit with renewed narrowing of the stream. When the pressure has been considerable, I have seen the vein become for a time wholly obstructed by the deposit. A similar deposit of lymph takes place in the artery. In one case, I observed that the artery, at the place pressed on, was actually not so much constricted as above and below, though, on account of the narrowness of the stream of blood from the presence of the lymphy deposit, it appears as much so at first sight.

Having subjected the web to the galvanic influence from a single pair of plates, I found all the smaller arteries of the part in a state of considerable tonic constriction, but the larger arteries constricted in a less degree. The effect of galvanism on the veins appeared to be to render their rythmical contractions somewhat more brisk, they having been previously rather languid. On cutting a vein across, I did not observe tonic constriction of it, any more than in the frog.

After the application of a drop of vinum opii to the web, the veins were found dilated as well as the arteries, and their rythmical contractions appeared to be suspended.

It has been stated, by an authority not liable to err, that, on mechanical irritation, both artery and vein of the bat's web gradually contract and close, and, by and by, dilate wider than before. And, again, that in bats, contraction of veins is quite aswell marked as that of arteries.

These statements, it will be observed, imply tonic contractility of the veins.

Notwithstanding my attention has been repeatedly directed to the point, I have not, as previously stated, been able to observe unequivocal evidences of tonic contractility of veins, in addition to their rythmical contractility. For this reason, I cannot help venturing on the supposition that Mr. Paget must have made his statements either from a hasty and imperfect observation of the proper rythmical contractions of theveins; or, seeing that in rythmical contraction of the veins, the constriction is never to closure, like that of the arteries, under some such misapprehension as to the nature of the vessel observed, as he certainly must have labored under when he supposed that arteries and veins of the second and third order open directly into each other without any intermedium of capillaries.

The arteries and their subdivisions anastomose freely with each other, forming a network all through the web, the meshes of which go on to diminish towards the free margin. Each artery, and each subdivision of an artery, is closely accompanied by a vein; and these veins, like the arteries they accompany, anastomose with each other. But it is to be remarked, that nowhere do the arteries and veins directly communicate. The only communication is the usual one through the medium of capillaries. The capillaries, the walls of which are destitute of contractility, received the blood from small arterial twigs, which arise from the arterial network, and return it to the venous radicles which open into corresponding veins. These arterial twigs, capillaries, and venous radicles, form networks within the meshes of the great vascular network, and a looped network at a margin of the web.

The observations recorded in the preceding pages were made principally with one-eighth of an inch object-glass, and the two lowest eyepieces, affording magnifying powers of 370 and 550 diameter.

The web of the wing was stretched out on the object-plate, wetted on both sides with water, and covered with a thin plate of glass at the spot to be examined.

*Appendix to the foregoing Paper.*

In consequence of the dark pigment in the cells of the epidermis of the web of the bat's wing, the structure of the vessels cannot be well made out except by dissection.

A small piece of the web containing vessels being detached and disposed in a drop of water, under the simple microscope, the two layers of skin may be readily torn from each other with needles, and the artery and vein, with their accompanying nerve, which lies between the two, separated in one bundle.

In pieces cut out of a web which had been dried, the bundle of vessels and nerve was, after tearing away the skin, left surrounded by a sheath of cellular and elastic fibres disposed longitudinally; but in pieces cut out from the living web and directly examined, this sheath was always detached along with the skin, and the vessels, with their accompanying nerve, at once laid bare.

Both artery and vein are seen to have a middle coat of circularly disposed muscular fibres; but the appearance of the fibres is different in the two vessels.

The fibres of the vein are about 1-3600dths of an inch broad, pale, grayish, semi-transparent, and granular-looking. In general aspect they very much resemble the muscular fibres of the lymphatic hearts of the frog. In none of the muscular fibres of the vein, however, did I detect an unequivocal appearance of transverse marking.

The fibres of the middle coat of the artery are not so pale-looking as those of the middle coat of the vein, are clearer, and exhibit a more strongly marked contour.

*Second Appendix.*

From a microscopical examination of the bloodvessels and circulation in the ears of the long-eared bat, I have ascertained that, different from what I discovered to be the case in the wings, the veins of the ears are unfurnished with valves, and are not endowed with rythmical contractility, and that the onward flow of blood in them is consequently uniform. I ought, perhaps, to qualify the statement that the veins of the ears are not endowed with rythmical contractility, by saying, that I think I noticed a very slight tendency to it here and there in a vein, but so slight as not to have the smallest effect on the flow of blood.

This observation regarding the ear of the bat illustrates how that the heart's action is sufficient of itself for the circulation of the blood in the body generally; but that being sufficient for that only, the supplementary force of rythmical contractility of veins, supported by the presence of valves, is called forth to promote the flow of blood in the wings, which on account of their extent, are, as regards their circulation, in a considerable degree, though not entirely, beyond the sphere of the heart's influence.

I may take this opportunity to mention, that I have also found the veins of the mesentery of the mouse destitute of rythmical contractility. —*Philos. Transact,* 1852.

